# RETRACTED: Abbas et al. Recent Developments in Artificial Super-Wettable Surfaces Based on Bioinspired Polymeric Materials for Biomedical Applications. *Polymers* 2022, *14*, 238

**DOI:** 10.3390/polym17182520

**Published:** 2025-09-18

**Authors:** Ansar Abbas, Chen Zhang, Muhammad Asad, Ahsan Waqas, Asma Khatoon, Sameer Hussain, Sajjad Husain Mir

**Affiliations:** 1 School of Chemistry, Xi’an Jiaotong University, Xi’an 710049, China; dr_ansar@stu.xjtu.edu.cn (A.A.); zhangchen@stu.xjtu.edu.cn (C.Z.); 2 Green Catalysis Center, College of Chemistry, Zhengzhou University, Zhengzhou 450001, China; muhammadasad2828@yahoo.com; 3 Key Laboratory of Applied Surface and Colloid Chemistry (Ministry of Education), School of Chemistry and Chemical Engineering, Shaanxi Normal University, Xi’an 710119, China; waqasahsan@snnu.edu.cn; 4 College of Business Administration, Imam Abdulrahman Bin Faisal University, Dammam 34212, Saudi Arabia; akhusain@iau.edu.sa; 5 School of Chemistry and Advanced Materials & BioEngineering Research (AMBER) Center, Trinity College Dublin, The University of Dublin, D02 PN40 Dublin, Ireland

The journal retracts the article titled “Recent Developments in Artificial Super-Wettable Surfaces Based on Bioinspired Polymeric Materials for Biomedical Applications” [1], cited above.

Following publication, concerns were brought to the attention of the publisher regarding an overlap between this publication [1] and a previously published review article [2], produced by a different author group.

Adhering to our procedure, an investigation was conducted by the Editorial Office and the Editorial Board that confirmed a significant level of overlap between these two publications [1,2]. Given the extent of the overlap, the Editorial Board have decided to retract this article as per MDPI’s retraction policy (MDPI—Research and Publication Ethics).

This retraction was approved by the Editor-in-Chief of the journal *Polymers*.

Authors agree to this retraction.
